# The complete chloroplast genome and phylogenetic analysis of *Jacobaea maritima* (Asteraceae)

**DOI:** 10.1080/23802359.2023.2238937

**Published:** 2023-07-22

**Authors:** Kai Zhang, Shoufu Gong

**Affiliations:** a Agricultural College, Xinyang Agriculture and Forestry University, Xinyang, China; b Horticultural College, Xinyang Agriculture and Forestry University, Xinyang, China

**Keywords:** *Jacobaea maritima*, chloroplast genome, horticulture plants, phylogeny

## Abstract

*Jacobaea maritima* is an important horticulture plant in the genus *Jacobaea.* Here, we assembled the complete chloroplast genome of *J. maritima*. The chloroplast genome was 153,857 bp in length, with a pair of inverted repeat regions (IRs) (27,936 bp) separated by a large single-copy region (LSC) (82,771 bp) and a small single-copy region (SSC) (15,214 bp). The complete chloroplast genome contained 112 unique genes, including 79 protein-coding genes, 29 tRNA genes, and 4 rRNA genes. The phylogenetic analysis showed *Jacobaea* was more closely related to *Senecio*, *Crassocephalum* and *Gynura*. The chloroplast genome of *J. maritima* can provide data to support future phylogenetic studies of *Jacobaea*.

## Background

*Jacobaea maritima* (L.) Pelser & Meijden belongs to the tribe Senecioneae branch in the family Asteraceae, and prefers to grow in cliffs and rocky coastal areas, usually distributed in the Mediterranean region, northwest Africa, southern Europe and western Asia, while in China it is mainly used as an ornamental plant (Galasso and Bartolucci 2015; Passalacqua et al. 2008). The *Jacobaea* was rich in secondary metabolites, including flavonoids, pyrrolizidine alkaloids, triterpenoids and aromatic acids (Maggio et al. 2015). Therefore, *Jacobae* species had become important for its ornamental and medicinal value and were grown in most areas with great climatic adaptability to different regions.

The phylogenetic study of *Jacobaea* is mainly based on plastid fragments and nuclear genes (Figure S1). In 2002, the molecular phylogeny of *Jacobaea* was studied to clarify species composition and interspecific relationships of *Jacobaea* (Pelser et al. 2002). A resolution of 60 representative species of the tribe Senecioneae based on DNA sequence data from the plastid sequences (*trn*T*-trn*L, *trn*L intron, *trn*K intron, and *matK* gene) and nuclear sequences (ITS1, 5.8S, and ITS2) revealed that *Jacobaea* was a strongly supported monophyletic group. The genera *Emilia*, *Packera* and *Pseudogynoxys* formed the sister clade of *Jacobaea*, but this relationship lacked strong bootstrap support. In 2003, the species composition and molecular phylogeny of *Jacobaea* were studied to identify the closest relatives of *J. vulgaris* (Pelser et al. 2003). Maximum parsimony and Bayesian inference analyses of DNA sequence data of the plastid (*trn*T-L, *trn*L intron, *trn*K intron, and *psbA-trn*H) and nuclear genome (ITS1, 5.8S, and ITS2) showed these markers to be suitable to assess the species composition of *Jacobaea*. In 2008, researchers performed phylogenetic analysis of the *J. maritima* group based on ITS1 sequence (Passalacqua et al. 2008). In 2021, a systematic study of diversity, polyploidy and morphology in the family Asteraceae based on single-copy nuclear genes, showed that the tribe Senecioneae was a monophyletic group and *Jacobaea* had a close relationship with *Senecio* and *Emilia* (Zhang et al. 2021).

However, studies on the chloroplast genome of *J. maritima* are still lacking, and phylogenetic questions regarding *Jacobaea* have not been addressed. In this study, we sequenced and assembled the chloroplast genome of *J. maritima* and performed comparative genomic analyses with the intention of further revealing the chloroplast genome characteristics of *Jacobaea* species. These results will provide a data base for phylogenetic studies of the tribe Senecioneae and *Jacobaea* species.

## Materials and methods

### Plant material, DNA extraction and sequencing

**Figure 1.**
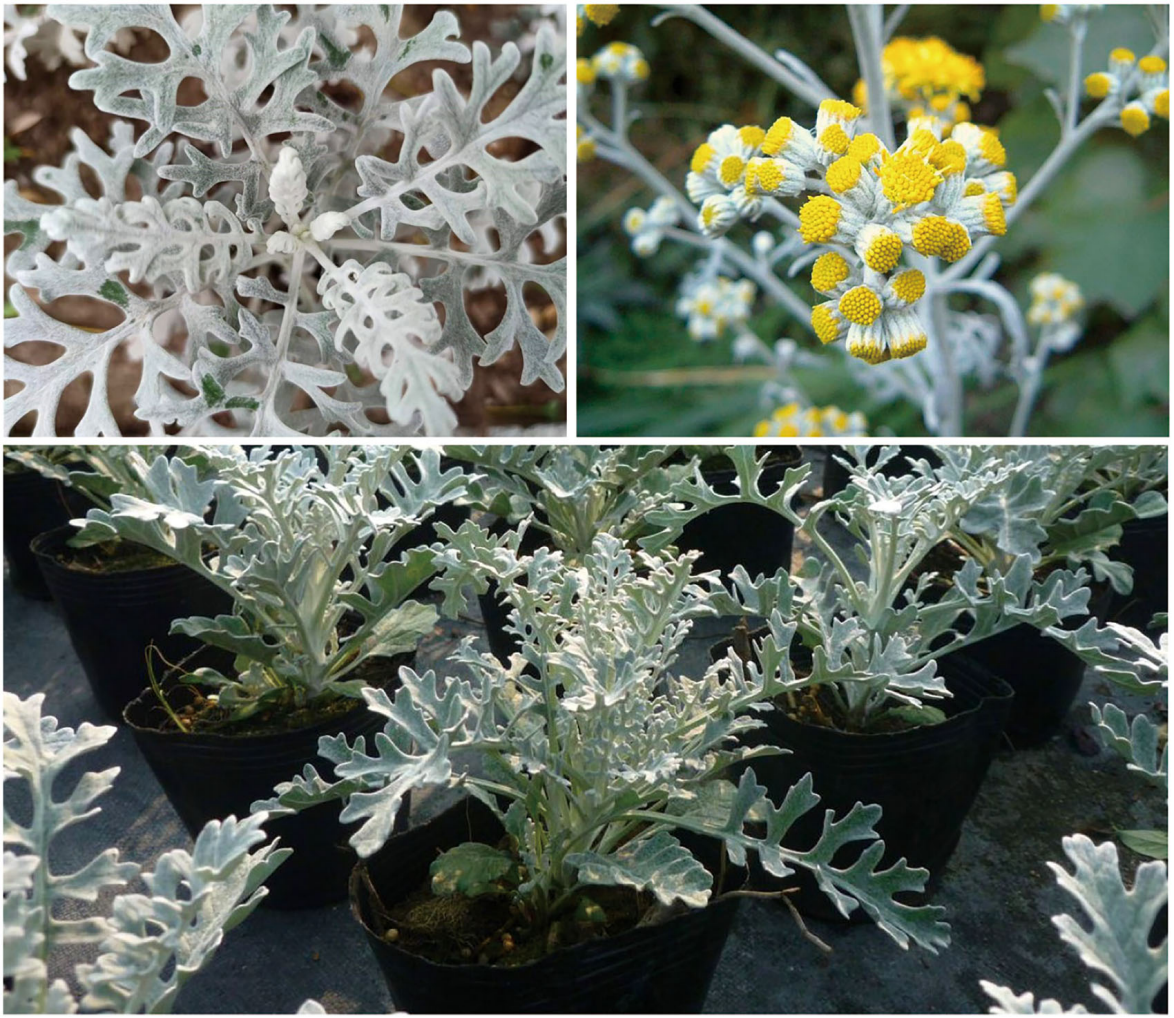
Reference images of *J. maritima* (in this study, this picture was taken by Kai Zhang). the voucher specimen was deposited in the herbarium of Xinyang Agriculture and Forestry University (voucher number: XJM22004; 114.21667° E, 32.28333° N).

Fresh leaves of *J. maritima* were sampled from second-year old plants in Xinyang, Henan, China (Figure 1; 114.21667° E, 32.28333° N). The voucher specimen was deposited in the herbarium of Xinyang Agriculture and Forestry University (voucher number: XJM22004; Mr. Gong, 2000230016@xyafu.edu.cn). The total genomic DNA was extracted using the HiPure Plant DNA Mini Kit (Magen, Guangzhou, China). A DNA library with an average length of 500 bp was constructed according to the instructions of the TruSeq DNA PCR-Free LT Sample Prep kit, followed by high-throughput sequencing (paired-end 250 bp) using the Illumina NovaSeq6000 platform. Raw reads were filtered using Trimmomatic v. 0.39 with default parameters retrieving the clean data.

### Genome assembly and annotation

De novo genome assembly from the clean data was accomplished using NOVOPlasty software (Dierckxsens et al. 2017), with the seed input file consisting of the protein-coding genes from the reference genome (*J. vulgaris*, GenBank: NC_015543) with *k*-mer setting of 39. Then we used samtools v1.7 (Li et al. 2009) and bedtools v2.28 (Quinlan and Hall 2010) for coverage depth detection, setting the window and step size to 200 bp, respectively. We also used MUMmer v4.0 (Marçais et al. 2018) for collinearity analysis. The chloroplast genome was annotated by using CPGAVAS2 (Shi et al. 2019), PGA (Qu et al. 2019) and Geneious Prime v. 2022.2.2 with a reference genome (*J. vulgaris*, GenBank: NC_015543). GB2sequin (https://chlorobox.mpimp-golm.mpg.de/GenBank2Sequin.html) was used to confirm the annotation result, and then CPGView (Liu et al. 2023) (http://www.1kmpg.cn/cpgview/) was used to test the accuracy of these genes. The genome map was drawn by Chloroplot (https://irscope.shinyapps.io/Chloroplot/).

### Repeat sequence and IR boundary analysis

The simple sequence repeats (SSRs) were identified using the online website MISA (https://webblast.ipk-gatersleben.de/misa/), including mono-, di-, tri-, tetra-, penta-, and hexa-nucleotides with minimum numbers of 10, 5, 4, 3, 3, and 3, respectively (Beier et al. 2017). Additionally, REPuter (https://bibiserv.cebitec.uni-bielefeld.de/reputer/) was used to calculate palindromic repeats, forward repeats, reverse repeats, and complementary repeats with the following settings: minimal repeat size of 30 bp (Kurtz et al. 2001). Furthermore, comparisons between the borders of the IR, SSC, and LSC regions were generated using IRscope (Amiryousefi et al. 2018).

### Phylogenetic analysis

To confirm the phylogenetic position of *J. maritima*, the chloroplast genomes of 20 Asteraceae species and one Brassicaceae species were downloaded from GenBank. The *Arabidopsis thaliana* was used as outgroup. We extracted 75 common protein-coding genes from the genome annotation files using PhyloSuite v. 1.2.2 (Zhang et al. 2020). Each protein-coding gene sequence was aligned using MAFFT v. 7.4 (Katoh and Standley 2013), and the 75 aligned sequences were concatenated. Based on the matrix of concatenate sequence, a phylogenetic tree was constructed using the maximum likelihood (ML) method implemented in IQ-TREE v. 2.1.2 (Nguyen et al. 2015), and the best model was inferred from ModleFinder (Kalyaanamoorthy et al. 2017). The bootstrap analysis was performed with 1000 replicates. Tree visualization was achieved in Figtree v. 1.4.3 (https://github.com/rambaut/figtree/releases).

## Results

### General features of the chloroplast genome

**Figure 2.**
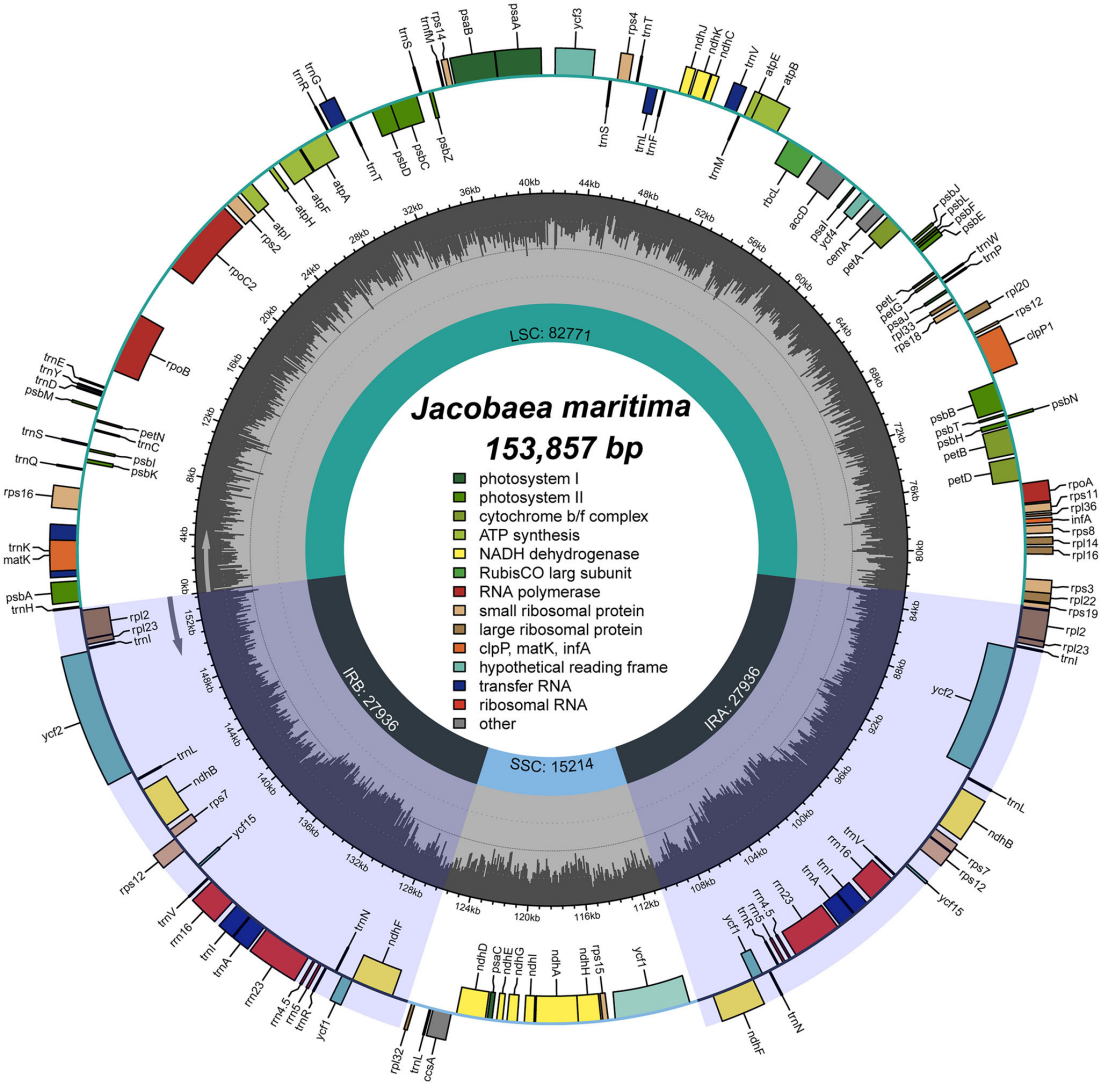
The chloroplast genome map of *J. maritima*. Genes on the inside of the circle are transcribed in a clockwise direction and genes on the outside of the circle are transcribed in a counter-clockwise direction.

We examined the chloroplast genome for completeness, coverage depth and collinearity, and the results showed that the chloroplast genome was reliable (Figure S2). Analysis of the genome annotation results also showed that the cis- and trans-splicing gene annotations were correct (Figure S3). The complete chloroplast genome of *J. maritima* was a typical circular tetrameric structure of 153,857 bp in length (Figure 2; Table 1; GenBank accession number: OL960706), consisting of a large single copy (LSC) region (82,771 bp), a small single copy (SSC) region (15,214 bp) and a pair of inverted repeats (IR) (27,936 bp). Percentages of the four base types for the whole chloroplast genome of *J. maritima* were 31.43% A, 31.43% T, 18.83% G, and 18.30% C. The chloroplast genome encoded 112 unique genes, including 79 protein-coding genes, 4 ribosomal RNA genes and 29 transfer RNA genes (Table S1). The chloroplast genome had a total GC content of 37.13% and the IR region had a GC content (41.37%) was significantly higher than that of the LSC region (35.43%) and the SSC region (31.81%). In the study, we detected 128 SSRs in the chloroplast genome of *J. maritima*, including 34 mononucleotides, 41 dinucleotides, 19 trinucleotides, 24 tetranucleotides, 7 pentanucleotides and 3 hexanucleotides (Table 1). Most SSRs were mononucleotides and dinucleotides, accounting for 58.59% of the total. In the chloroplast genomes of *J. maritima* and *J. vulgaris*, SSRs were most abundant in the LSC region and least in the IR region, and mainly concentrated in the coding region, with fewer SSRs in the non-coding region (Table 1; Table S2). This phenomenon may be related to the sequence conservativeness and GC content of different regions, etc. In addition, we detected 4 interspersed repeats in the chloroplast genome of *J. maritima*, including one forward repeat and 3 palindromic repeats.

**Table 1.** Summary of the chloroplast genomes of two Jacobaea species.

	Characteristic	*Jacobaea maritima*	*Jacobaea vulgaris*
Chloroplast genome	Size (base pair, bp)	153,857	150,689
	LSC length (bp)	82,771	82,855
	SSC length (bp)	15,214	18,276
	IR length (bp)	27,936	24,779
	Number of genes	112	112
	Protein-coding genes	79	79
	rRNA genes	4	4
	tRNA genes	29	29
	Total GC content	37.13%	37.32%
	LSC GC content	35.43%	35.40%
	SSC GC content	30.81%	30.51%
	IR GC content	41.37%	43.02%
	all SSR number	128	121
	LSC SSR number	110	96
	SSC SSR number	18	21
	IR SSR number	0	4

### IR boundaries analysis

In general, the length variation of the LSC/SSC region was lower than that of the IRa/IRb region. The chloroplast genome of *J. maritima* showed the expansion of the IR region and contraction of the SSC region compared to the chloroplast genome of *J. vulgaris* (Figure S4). In the chloroplast genome of *J. maritima*, *rps15* and *rpl32* were located within the SSC/IR boundary, and *ndhF* and *rpl2* were located outside the SSC/IR boundary. In contrast, in the chloroplast genome of *J. vulgaris*, *ycf1* was located within the SSC/IR boundary, *ndhF* was located within the SSC/IR boundary, and *rpl2* and *trn*N were located outside the SSC/IR boundary.

### Phylogenetic analysis

**Figure 3.**
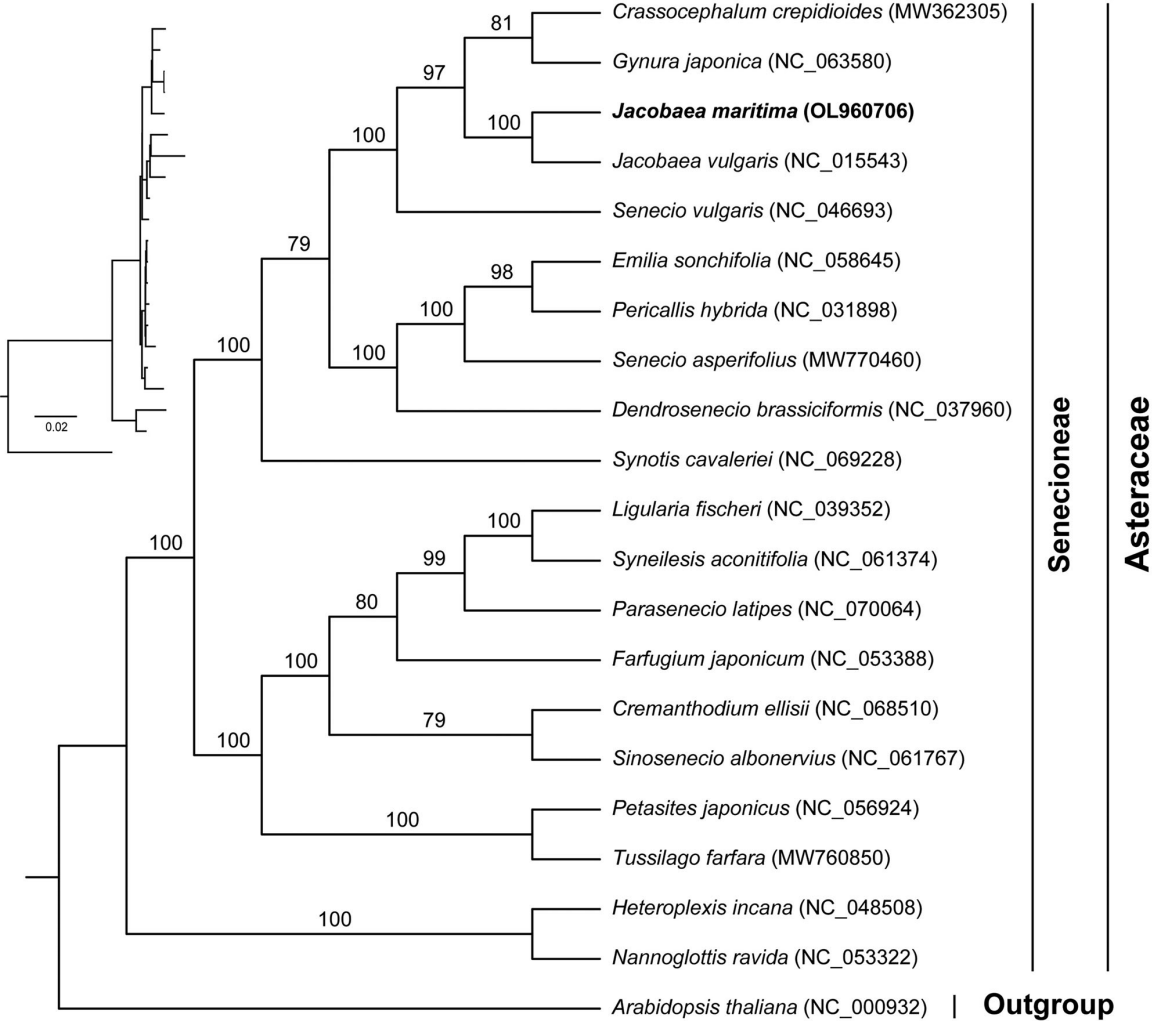
Phylogenetic tree based on the concatenated sequences of 75 common protein-coding genes in 21 species using maximum-likelihood (ML) method. The value above the branch indicates the bootstrap value.

To understand the phylogenetic position of *J. maritima* in the Asteraceae, we performed a phylogenetic analysis. The maximum-likelihood tree was constructed using IQ-TREE v. 2.1.2 (Nguyen et al. 2015) with the best-fit model of TVM + F+R4. The phylogenetic analysis showed high bootstrap for most of nodes in the phylogenetic tree, showing the reliability of the phylogeny (Figure 3). Our results clearly showed that *Jacobaea* was more closely related to *Senecio*, *Crassocephalum* and *Gynura*.

## Discussion

Compared with nuclear and mitochondrial genomes, chloroplast genomes are highly conserved and have been widely used in phylogenetic and evolutionary studies. With the development of high-throughput sequencing technology, chloroplast genome sequences play an important role in phylogenetic study (Li et al. 2021). In this study, the chloroplast protein-coding genes tree showed high support with credibility. Our phylogenetic results were also in general agreement with the previous results (Figure S1) (Passalacqua et al. 2008; Zhang et al. 2021), which showed that *Jacobaea* was more closely related to *Senecio*, *Crassocephalum* and *Gynura*. Future enrichment of chloroplast genomic data of *Jacobaea* species is needed to further resolve intra-generic phylogenetic relationships. However, these results are limited due to the matrilineal inheritance of the chloroplast genome (Krawczyk et al. 2018). An accurate phylogenetic relationship still requires an integrated analysis of the nuclear and organelle genomes (Górniak et al. 2010). More molecular data is needed in the future to determine the relationships between *Jacobaea* and other genus in the family Asteraceae.

## Conclusion

In this study, the chloroplast genome of *J. maritima* was *de novo* assembled with short reads. This chloroplast genome had a typical tetrameric structure similar to that of most angiosperms. In addition, the phylogenetic tree strongly supported the phylogenetic position of *J. maritima,* showing that *Jacobaea* was more closely related to *Senecio*, *Crassocephalum* and *Gynura*. Thus, the chloroplast genome of *J. maritima* not only enriches the genomic information of *Jacobaea*, but also lays the foundation for understanding the evolution of Asteraceae species.

## Supplementary Material

Supplemental Material

Supplemental Material

Supplemental Material

Supplemental Material

Supplemental Material

Supplemental Material

## Data Availability

The contact person of the specimen is Shoufu Gong (2000230016@xyafu.edu.cn). The genome sequence data that support the findings of this study are openly available in GenBank of NCBI at (https://www.ncbi.nlm.nih.gov/) under the accession no. OL960706. The associated BioProject, SRA, and Bio-Sample numbers are PRJNA775801, SRX12813512, and SAMN22625029 respectively.
