# Supplementary figures and images for: The complete chloroplast genome and phylogenetic analysis of Jacobaea maritima (Asteraceae)
Source: Mitochondrial DNA B Resour. 2023 Jul 22;8(7):771–6. doi: 10.1080/23802359.2023.2238937 (PMC10364569; doi:10.1080/23802359.2023.2238937)

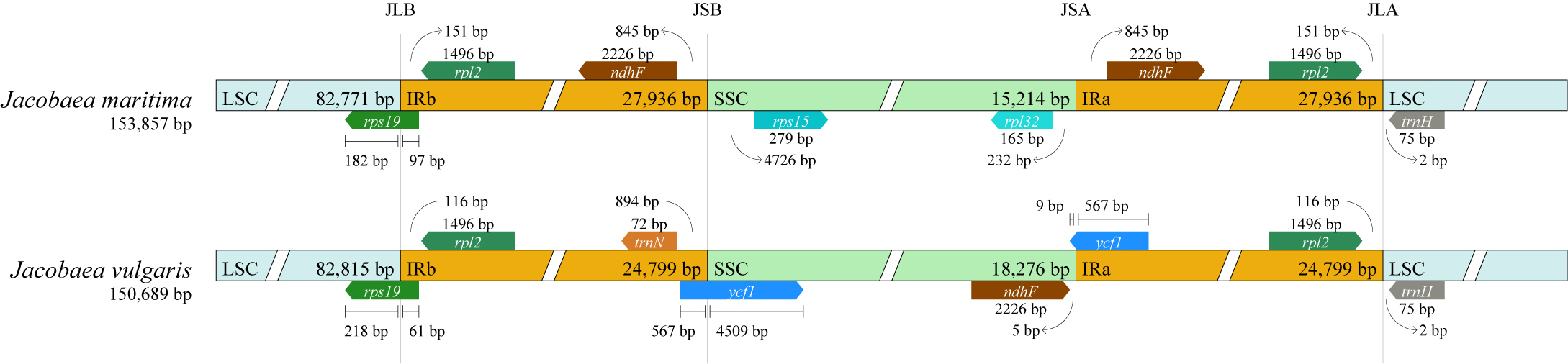

Supplement: Supplemental Material [file TMDN_A_2238937_SM9647.jpg]

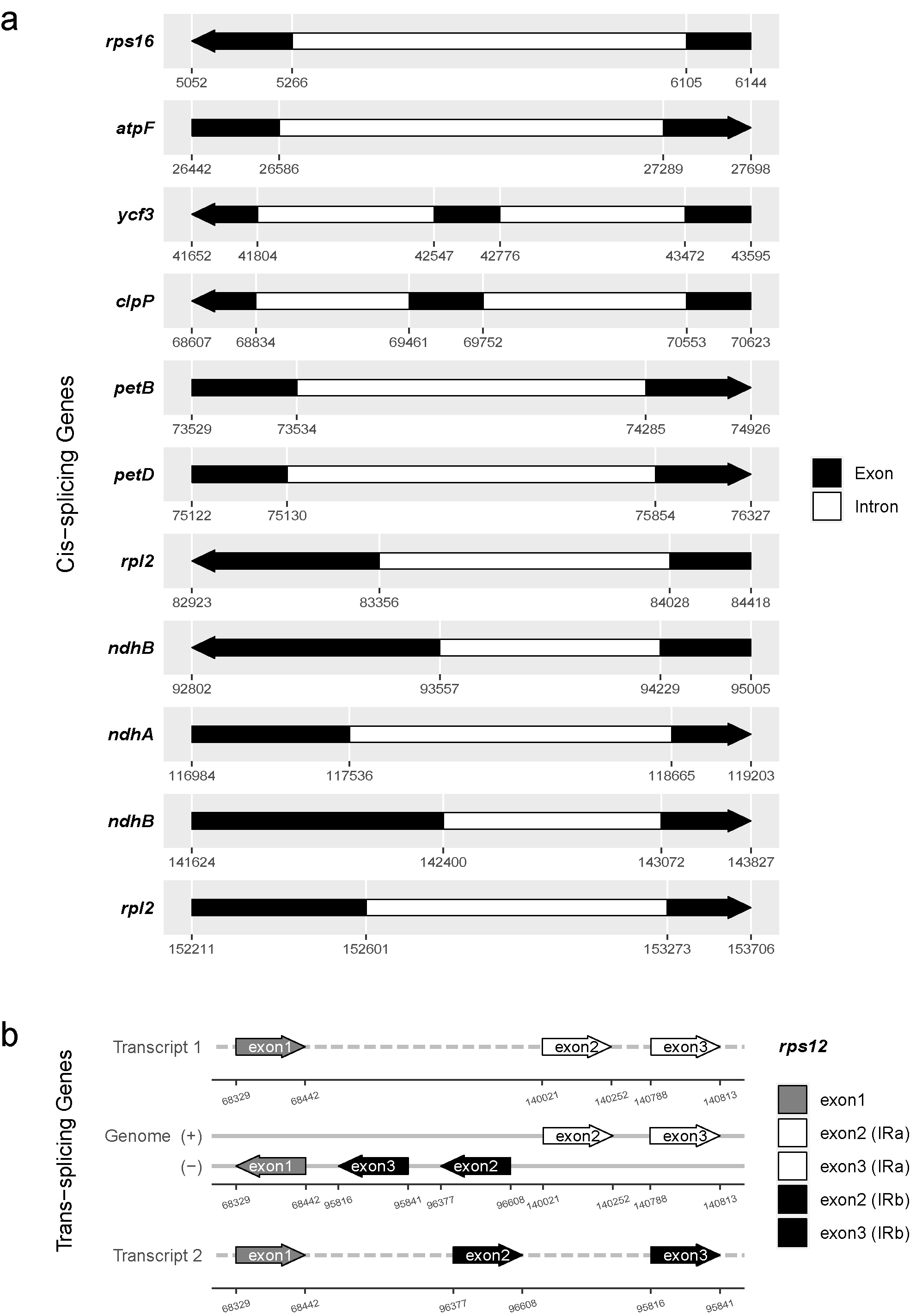

Supplement: Supplemental Material [file TMDN_A_2238937_SM9640.jpg]

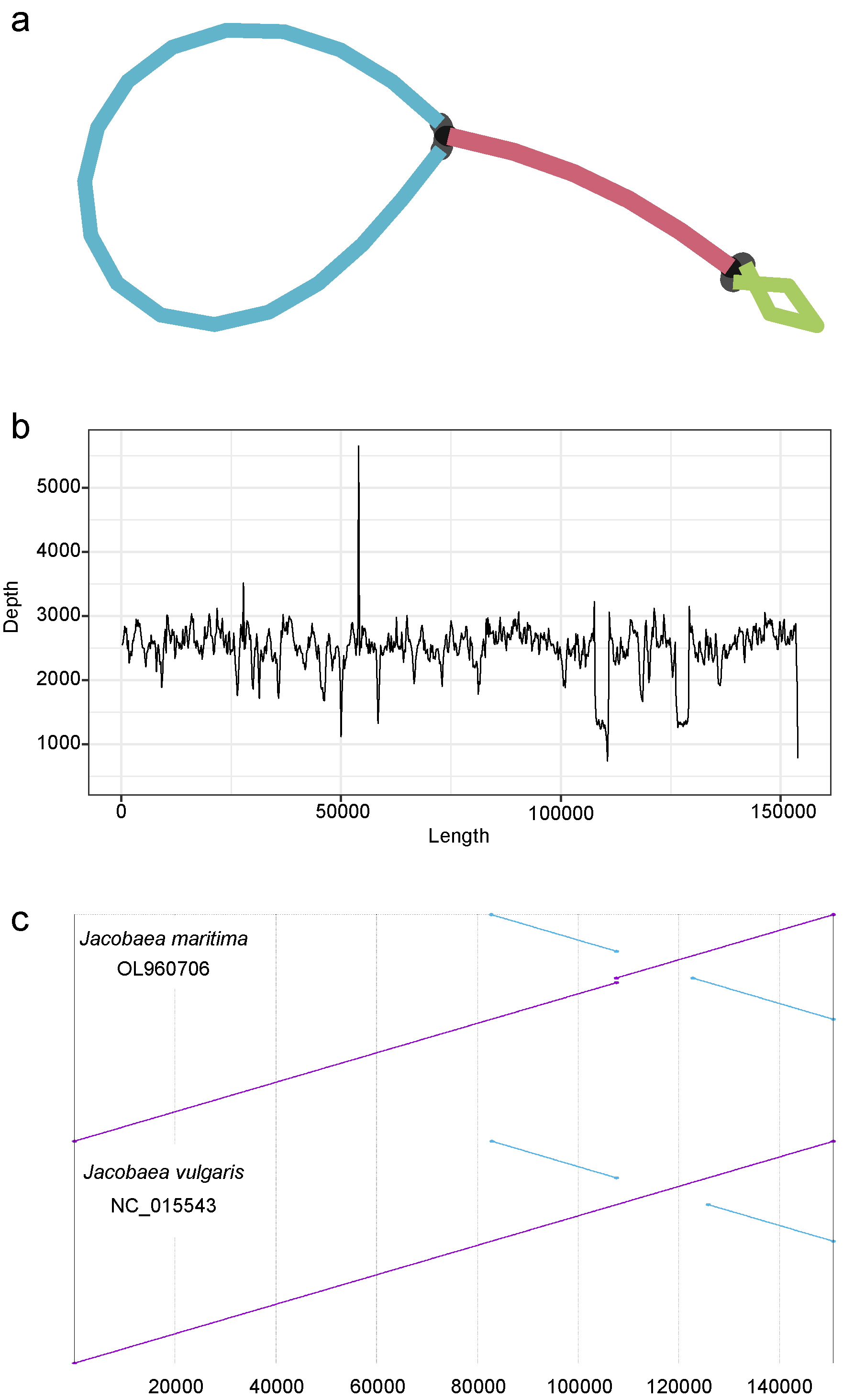

Supplement: Supplemental Material [file TMDN_A_2238937_SM9632.jpg]

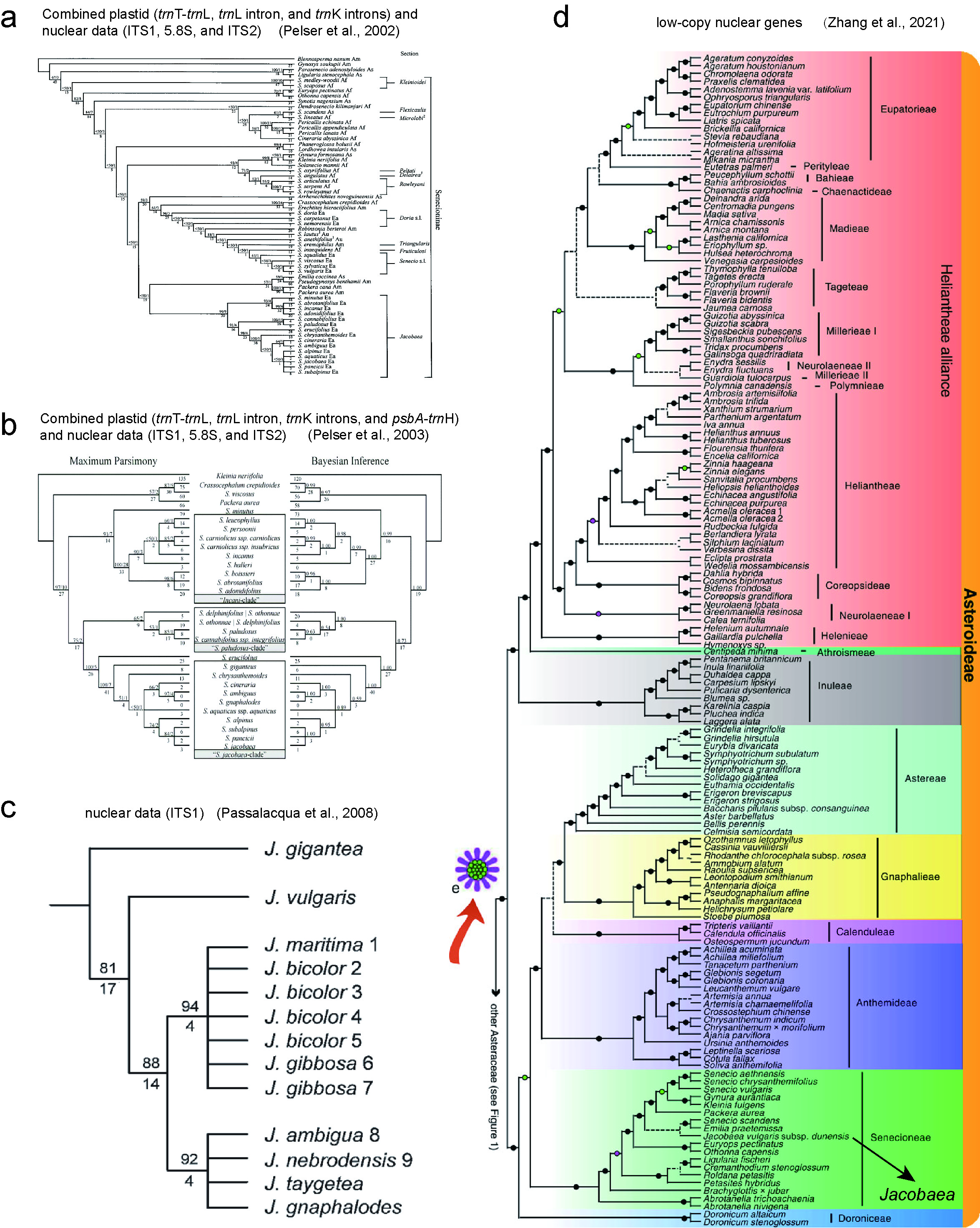

Supplement: Supplemental Material [file TMDN_A_2238937_SM9616.jpg]
